# Viral speciation through subcellular genetic isolation and virogenesis incompatibility

**DOI:** 10.1038/s41467-020-20575-5

**Published:** 2021-01-12

**Authors:** Vorrapon Chaikeeratisak, Erica A. Birkholz, Amy M. Prichard, MacKennon E. Egan, Avani Mylvara, Poochit Nonejuie, Katrina T. Nguyen, Joseph Sugie, Justin R. Meyer, Joe Pogliano

**Affiliations:** 1 Division of Biological Sciences, University of California, San Diego, CA 92093 USA; 2 Department of Biochemistry, Faculty of Science, Chulalongkorn University, Bangkok, 10330 Thailand; 3 Institute of Molecular Biosciences, Mahidol University, Salaya, Nakhon Pathom Thailand

**Keywords:** Molecular evolution, Bacteriophages, Phage biology

## Abstract

Understanding how biological species arise is critical for understanding the evolution of life on Earth. Bioinformatic analyses have recently revealed that viruses, like multicellular life, form reproductively isolated biological species. Viruses are known to share high rates of genetic exchange, so how do they evolve genetic isolation? Here, we evaluate two related bacteriophages and describe three factors that limit genetic exchange between them: 1) A nucleus-like compartment that physically separates replicating phage genomes, thereby limiting inter-phage recombination during co-infection; 2) A tubulin-based spindle that orchestrates phage replication and forms nonfunctional hybrid polymers; and 3) A nuclear incompatibility factor that reduces phage fitness. Together, these traits maintain species differences through Subcellular Genetic Isolation where viral genomes are physically separated during co-infection, and Virogenesis Incompatibility in which the interaction of cross-species components interferes with viral production.

## Abstract (web summary)

Virus speciation cannot be fully explained by the evolution of different host specificities. Here, Chaikeeratisak et al. identify ways viruses can remain genetically isolated despite co-infecting the same cell, providing insight into how new virus species evolve.

## Introduction

Bacteriophages are the most abundant microbes on Earth and arguably harbor the most genetic diversity of any taxonomic group^1,2^. Viruses are known to evolve quickly due to their large population sizes, short generation times, and frequently high mutation rates. Mutations can become fixed in the population by strong selective forces or by drift. Until recently, it was thought that this genetic diversity was relatively unstructured with phages freely exchanging genes^3–5^. Through analyses of a growing number of full phage genome sequences, it has become clear that the diversity coalesces into biological species clusters where more genetic information is exchanged within, rather than between clusters^6^. This observation leads to the question of how phages evolve barriers to genetic exchange. Adaptation to different hosts has been shown to cause viral speciation since viruses recombine when multiple particles infect the same cell. Evolving divergent host specificities therefore has the side effect of developing barriers to genetic exchange^7–9^. However, the evolution of host specificities cannot fully explain viral speciation because viruses that infect the same hosts sometimes form genetically isolated species^6,10^. Viral traits must have evolved that form genetic barriers during co-infection of the same cell and could ultimately lead to viral speciation. In the study of two closely related jumbo phages that infect *Pseudomonas aeruginosa*, we hypothesized that specific viral mechanisms must exist that contribute to genetic isolation. Here we sought to identify potential barriers to viral genetic exchange and quantitate the extent to which they contribute to reproductive isolation. Our approach is analogous to those historically performed on eukaryotes where the reproductive isolation caused by different phenotypic and genetic characteristics is quantified^11^.

During infection of *Pseudomonas*, jumbo phages 201Φ2-1, ΦPA3, and ΦKZ establish an intricate subcellular organization that we reasoned may impact gene flow^12–16^. These jumbo phages replicate by enclosing their DNA within a proteinaceous shell that forms a nucleus-like compartment, separating enzymes involved in DNA replication, transcription, and repair from ribosomes and metabolic enzymes in the cytoplasm^14,15^. A tubulin-like protein, PhuZ, assembles a spindle early during lytic growth that plays multiple roles during the life cycle of the phage^12–17^. At early stages of infection, the PhuZ spindle uses dynamic instability to position the phage nucleus at midcell^12–15^. Later during infection, the spindle uses treadmilling to traffic newly assembled capsids through the cell and to distribute them around the nucleus^16^. Nonfunctional spindles result in severe nucleus mispositioning, loss of capsid trafficking, and a 50% decrease in phage progeny^12–16^. Given the organizational complexity of these phages, it was unclear how they might interact with one another during co-infection of a single cell when there is an opportunity for genetic exchange. Here we characterize co-infecting phages and identify two types of barriers to gene flow. We show that Subcellular Genetic Isolation occurs when a nucleus-like compartment physically separates viral genomes during co-infection. We also show that co-infections by different viruses can be less productive because of incompatibility between divergent viral components. This Virogenesis Incompatibility blocks gene flow by reducing the chance of producing recombinant progeny.

## Results

**Fig. 1:**
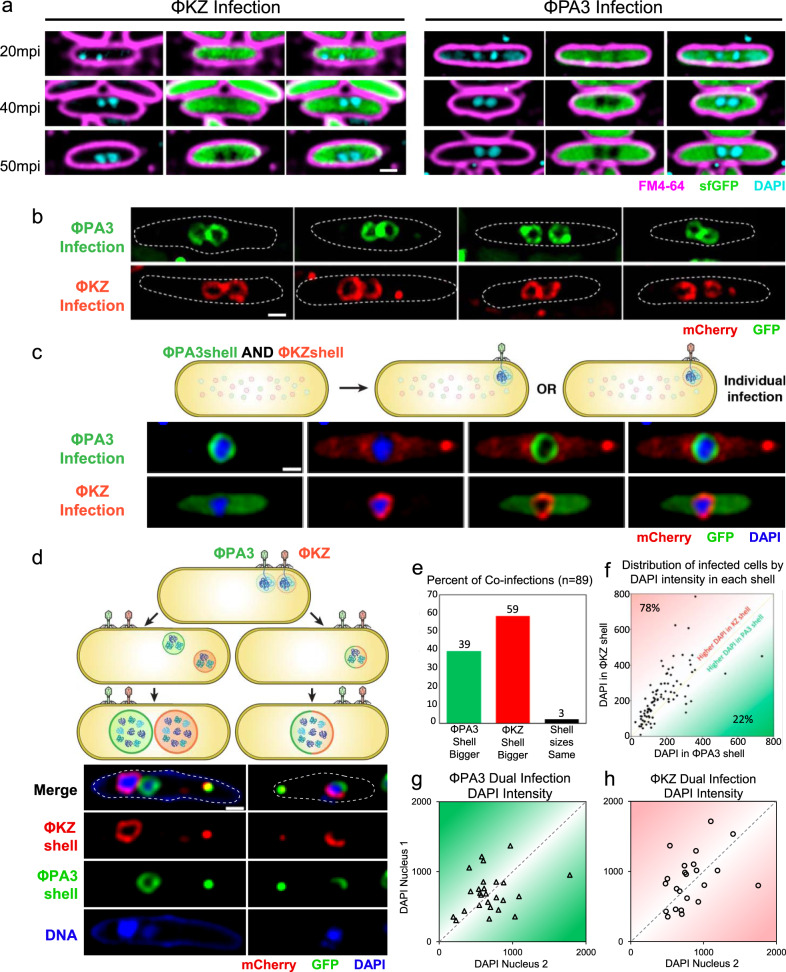
Subcellular Genetic Isolation occurs between identical and divergent co-infecting phages. **a** *P. aeruginosa* infected for 50 min with either ΦPA3 or ΦKZ frequently harbors more than one phage nucleoid. Cell membranes (magenta), DNA (cyan), GFP (green). **b** GFP-tagged ΦPA3 shell (green) and mCherry-tagged ΦKZ shell (red) reveals two nucleoids separated by nuclear shells at 50 mpi. **c**, **d** Cells expressing both GFP-ΦPA3shell and mCherry-ΦKZshell and were infected with either ΦPA3 or ΦKZ (**c**) or both phages (**d**). **c** GFP-ΦPA3shell only forms a shell (green) when infected with ΦPA3 and mCherry-ΦKZshell only forms a shell (red) when infected with ΦKZ. Phage DNA (blue). **d** Co-infected cells formed two separate red and green nuclei (~75%) or one nucleus with both red and green shell components (~25%). **e**–**h** *n* = 89 co-infected cells, **e** Percentage of co-infected cells with a larger ΦPA3 or ΦKZ shell. **f** Distribution of co-infected cells based on DAPI intensity inside shells. **g**, **h** Distribution of single-species co-infecting nuclei by DAPI intensity for ΦPA3 or ΦKZ, respectively. Using a cut off of at least 5% difference between the two values to establish if they are different from one another shows that 88% of the nuclei have unequal DNA content. Source data are provided as a Source Data file. Scale bars in panel **a**–**d** equals 1 μm.

We expected that two identical phages infecting one cell could either form a single nucleus that would facilitate inter-phage recombination or two separate nuclei, greatly reducing gene exchange. We first used DAPI staining to visualize phage DNA and soluble GFP to visualize the cytoplasm, and found that cells infected with either ΦPA3 or ΦKZ frequently (24%, *n* = 156 and 19%, *n* = 368 at 20 min post-infection, mpi; 21%, *n* = 142 and 18%, *n* = 307 at 50 mpi, respectively; Fig. S1A) formed two separate phage nucleoids in a single cell (Fig. 1a). The similar percentage of cells with more than one nucleoid at 20 mpi and 50 mpi suggests that the nucleoids remained separated throughout the infection. To confirm that these nucleoids were in separate compartments, we fluorescently tagged the ΦPA3 shell protein with GFP and the ΦKZ shell with mCherry and followed nucleus assembly during infection (Fig. 1b). Each fluorescent protein-shell fusion formed diffuse fluorescence and small foci when uninfected (Fig. S1B) and assembled a nuclear shell during infection (Fig. 1b). Direct visualization of each nucleus confirmed that a significant fraction of infected host cells contained two nuclei (ΦPA3, 18%, *n* = 104; ΦKZ, 14%, *n* = 117; Fig. 1b). In cells containing two distinct nuclei, both were adjacent to one another and positioned at midcell by the PhuZ spindle (Fig. 1b). These results demonstrate that during single-species co-infections, separate replication compartments for identical genomes can be established within the same cell. These data also suggest that the phage nucleus forms a physical barrier that separates co-replicating viral genomes, thereby reducing the potential for genetic exchange even between two identical genomes. We refer to the separation of co-replicating viral genomes by physical or spatial barriers, such as the phage nucleus, as Subcellular Genetic Isolation. This phenomenon has already been observed in Herpes simplex virus 1 and Poxvirus which form spatially separated replication factories^18,19^. Recombination does not occur unless the replication factories coalesce^18,19^. Traits that cause Subcellular Genetic Isolation create a barrier to genetic exchange which allows for evolutionary divergence and the accumulation of reproductive incompatibilities that arise by neutral drift or other processes. Subcellular Genetic Isolation is therefore a potentially common mechanism of viral speciation.

To further test the theory that the phage nucleus results in Subcellular Genetic Isolation, we visualized co-infections of ΦPA3 and ΦKZ, two related phages whose shells share ~45% amino acid identity. We expressed both fluorescently tagged shell proteins from a single plasmid and determined if assembly into a nucleus was promiscuous or specific to the cognate phage from which the shell originated (Fig. 1c). We found that each shell protein only assembled into a nuclear shell containing DNA during infection with the cognate phage (Fig. 1c) and therefore can be used to track individual phage nuclei during co-infection of a single cell.

Next, we followed the fate of the two different phages during co-infection of *P. aeruginosa* expressing both fluorescently tagged shell proteins, by infecting it simultaneously with ΦPA3 and ΦKZ (Fig. 1d). Approximately 25% (*n* = 295) of doubly infected cells contained shell structures that incorporated components from each species (Fig. 1d, half red and half green shell), whereas the majority (75%; *n* = 295) of co-infections resulted in separate red and green shells that physically separated the genomes (Fig. 1d). Thus, during co-infections resulting from either a single species (Fig. 1a, b) or two species (Fig. 1d), phage can assemble separate nuclei, supporting the idea that the phage nucleus is a speciation factor that creates a physical barrier which separates co-replicating viral genomes and establishes Subcellular Genetic Isolation.

A second mechanism that is likely universal among viruses and that can contribute to genetic isolation, even without the formation of nuclear shells, is intracellular competition between speciating strains. If one strain of virus gains an advantage over the other, then it will reproduce more within cells and it will be more likely to recombine with conspecific genomes. The probability of cross-species recombination can be predicted by the rate at which genomes encounter each other, which, without subcellular organization or other factors that influence recombination rates, is equal to their relative genome frequencies (Fig. S2). When each strain is equally competitive, then half of all recombinations will occur within the same strain and the other half between strains. As competitive differences increase between strains, their relative frequencies will shift, and the amount of cross-species recombination will equal two times the product of each strains’ frequency (Fig. S2). Phages ΦKZ and ΦPA3 were added simultaneously to cells, yet after 1 h, one phage nucleus was almost always (97%, *n* = 89) larger than the other (Fig. 1e), demonstrating competition between ΦKZ and ΦPA3. DAPI staining was used to estimate relative DNA content by integrated intensity within individual phage nuclei. We found a direct correlation between nucleus size and amount of phage DNA (Fig. S1E, F). ΦKZ outcompetes ΦPA3 in 78% (*n* = 89) of co-infections based on DNA content (Fig. 1f). For comparison, we quantitated DAPI staining during co-infection by a single species and found that one phage usually (~88%; *n* = 50) out-replicated the identical phage, suggesting that competition is inherent during co-infections (Fig. 1g, h). We measured the relative genome frequencies of ΦPA3 (q) and ΦKZ (p) based on DAPI staining for each co-infected cell, and compared the expected probability of cross-species recombination, 2pq, to the probability that two genomes present at equal frequencies would recombine (when *p* = *q*, 2*pq* = 0.5) (Fig. S2). For ΦPA3 and ΦKZ co-infections, the average 2*pq* value was 0.47, which corresponds to a 6 percent reduction of cross-species recombination (Fig. S2). Intracellular competition does not have much of an impact on limiting interspecific recombination for these species; however, the effect is predicted to magnify as competitive differences increase (Fig. S2). Competition between viruses may have an even more important long-term effect on speciation than calculated above since inter-strain competition can trigger a coevolutionary arms race that will drive further genetic divergence and help reinforce species separation.

**Fig. 2:**
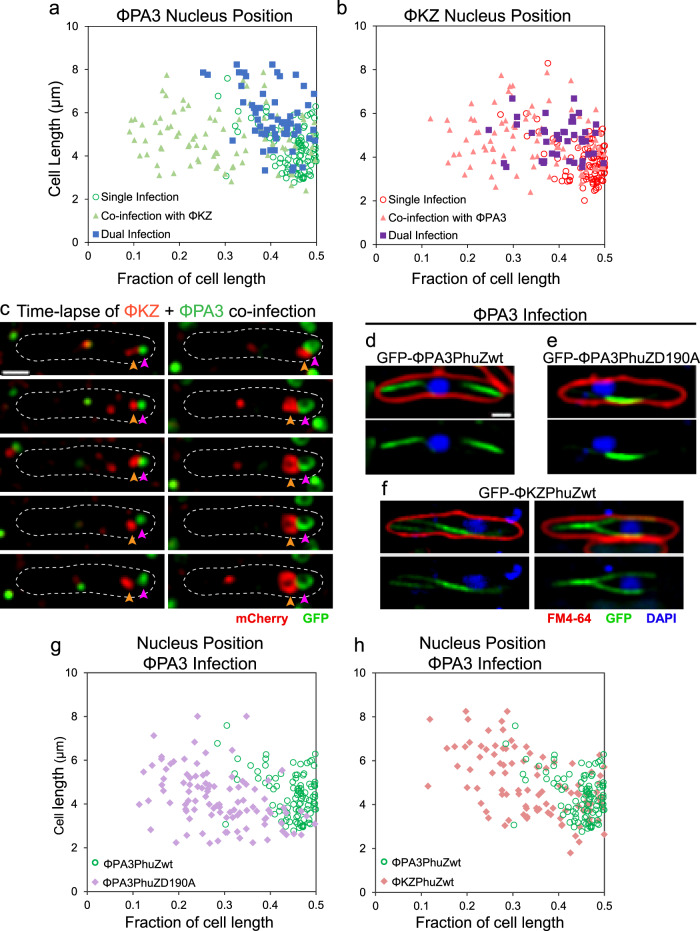
Nuclei are mispositioned during cross-species co-infections. **a**–**c** Cells expressing both GFP-ΦPA3shell (green) and mCherry-ΦKZshell (red) were infected with either ΦPA3 or ΦKZ or both. **a** ΦPA3 nucleus position when singly infected with ΦPA3 (open circles, *n* = 100), when dual infected with ΦPA3 (solid squares, *n* = 56) resulting in two ΦPA3 nuclei in a single cell, or when co-infected with ΦKZ (solid triangles, *n* = 89). **b** ΦKZ nucleus position when singly infected with ΦKZ (open circles, *n* = 100), when dual infected with ΦKZ (solid squares, *n* = 40) resulting in two ΦKZ nuclei in a single cell, or when co-infected with ΦPA3 (solid triangles, *n* = 89). **c** Time-lapse (sec, seconds) of co-infecting nuclei failing to migrate to midcell (mpi, minutes post-infection). **d**–**f** Cells expressing wildtype sfGFP-ΦPA3PhuZ (**d**), mutant sfGFP-ΦPA3PhuZD190A (**e**), or wildtype sfGFP-ΦKZPhuZ (**f**) showing the nucleus (blue, DAPI) and spindle (green, GFP), cell membrane (red, FM4-64) when infected with ΦPA3. **g**, **h** ΦPA3 nucleus position displayed as fraction of cell length for cells expressing wildtype sfGFP-ΦPA3PhuZ (**g**, **h**, green, *n* = 100), mutant sfGFP-ΦPA3PhuZD190A (**g**, purple, *n* = 100), or wildtype sfGFP-ΦKZPhuZ (**h**, pink, *n* = 100). Scale bar in panel (**d**) equals 1 μm and all panels in (**c**–**f**) are at the same scale. Data for ΦPA3 nucleus position is repeated in graphs (**a**, **g**, & **h**) for reference. Source data are provided as a Source Data file.

**Fig. 3:**
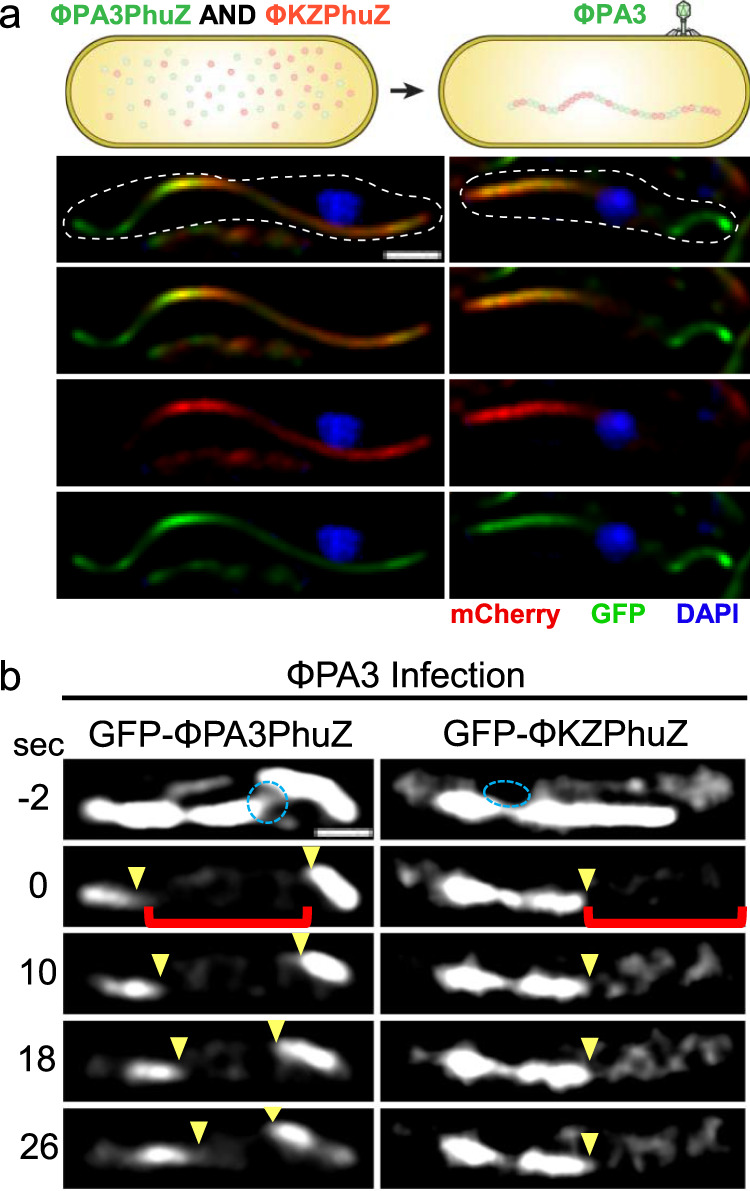
Nonfunctional hybrid spindles form through cross-species mixing of PhuZ monomers. **a** Simultaneous expression of sfGFP-ΦPA3PhuZ (green) and mCherry-ΦKZPhuZ (red) results in hybrid filaments (yellow) and mispositioning of the ΦPA3 nucleus (blue). **b** Photobleaching of sfGFP-ΦPA3PhuZ (left) shows movement of bleached zones indicating treadmilling (yellow arrows) during ΦPA3 infection. Photobleaching of hybrid spindles formed by expressing sfGFP-ΦKZPhuZ and cross infecting with ΦPA3 demonstrates filaments are not dynamic. Scale bars equal 1 μm.

In eukaryotes, reproductive isolation can be driven by pre- or post-zygotic incompatibilities between gametes, limiting the ability to form offspring. While viruses do not form gametes or zygotes, our co-infection experiments revealed that the PhuZ spindle is an incompatibility factor that limits the production of phage offspring due to deleterious interactions between cross-species alleles (Figs. 2, 3). Normally, the phage nucleus is positioned at midcell by the spindle within 1 h post-infection for single or double infections by one species of phage (Figs. 1a–c and 2a, b, d)^12–15^. However, in co-infections with both ΦPA3 and ΦKZ, the two nuclei were always adjacent to one another and usually (92%, *n* = 89) mispositioned (Figs. 1d and 2a–c). Normally, >95% of nuclei occur within 20% of the cell midpoint at 60 mpi^12–15^. Mispositioning results in a large percentage of nuclei outside of the 20% boundary^12–15^. In time-lapse microscopy (Fig. 2c), two small nuclei at the cell pole grew in size over time but failed to be moved to the center. This loss of positioning suggests that spindles are nonfunctional during co-infections. One possibility is that each phage establishes a separate bipolar spindle that cannot properly position two adjacent nuclei. Alternatively, a single nonfunctional spindle forms by co-assembly of the divergent PhuZ monomers which share only 37% sequence identity^15^. To understand the molecular basis of nuclear mispositioning, we expressed GFP fusions to either ΦPA3 PhuZ or ΦKZ PhuZ at low levels below the critical threshold for filament assembly^15^, and followed spindle assembly and dynamics during cognate or cross-infection with ΦPA3. When ΦPA3 infected cells expressing the cognate sfGFP-ΦPA3PhuZ, a properly functioning bipolar spindle formed, flanking a centrally located phage nucleus as expected (Fig. 2d, g, green). However, when ΦPA3 infected cells expressing sfGFP-ΦKZPhuZ (Fig. 2f, h) or untagged ΦKZPhuZ (Fig. S3), the spindles were malformed, usually resulting in one long static filament within the cell (Fig. 2f), and the nuclei were as severely mispositioned (Fig. 2h, pink) as in cells expressing a ΦPA3PhuZD190A catalytic mutant^15^ that renders the spindle inactive (Fig. 2e, g, purple). This suggests that the two divergent PhuZ monomers interfere with one another by co-assembling in a non-productive manner.

To directly observe if these proteins can co-assemble, we simultaneously expressed both mCherry-ΦKZPhuZ and sfGFP-ΦPA3PhuZ proteins in *P. aeruginosa*. Upon infection with ΦPA3, instead of forming a dynamic bipolar spindle as occurs when expressing sfGFP-ΦPA3PhuZ alone (Fig. 2d;^15^), hybrid spindles composed of both ΦPA3 PhuZ and ΦKZ PhuZ monomers were formed, and the nuclei were mispositioned (Fig. 3a). Fluorescence recovery after photobleaching (FRAP) demonstrated that normal PhuZ spindles treadmill (Fig. 3b, left;^16^), while the hybrid spindles were not dynamic (Fig. 3b, right). Nuclei are mispositioned during co-infections of cells that do not express GFP-tagged PhuZ proteins (Fig. S3). Taken together, these results demonstrate that PhuZ monomers from the two phages co-assemble nonproductively, disrupting the dynamic properties of the spindle. Given the central roles of the PhuZ spindle in nucleus centering^12–15^, nucleus rotation^14,16^, and capsid transport for DNA packaging^16^, the nonfunctional hybrid spindles suggest that PhuZ is a speciation factor that limits the ability of these two phages to reproduce while co-infecting a single host cell. Analogous to pre-zygotic incompatibilities experienced by eukaryotes where combinations of alleles prevent the production of hybrid offspring, hybrid spindles present an incompatibility that limits the production of progeny during the process of viral replication. We term this Virogenesis Incompatibility, in which viral encoded factors create barriers to the successful production of two different viruses within the same cell. Since loss of spindle function causes a 50% decrease in phage production^12^, we estimate that the Virogenesis Incompatibility caused by hybrid spindles results in a 50% reduction in progeny phage during co-infection.

**Fig. 4:**
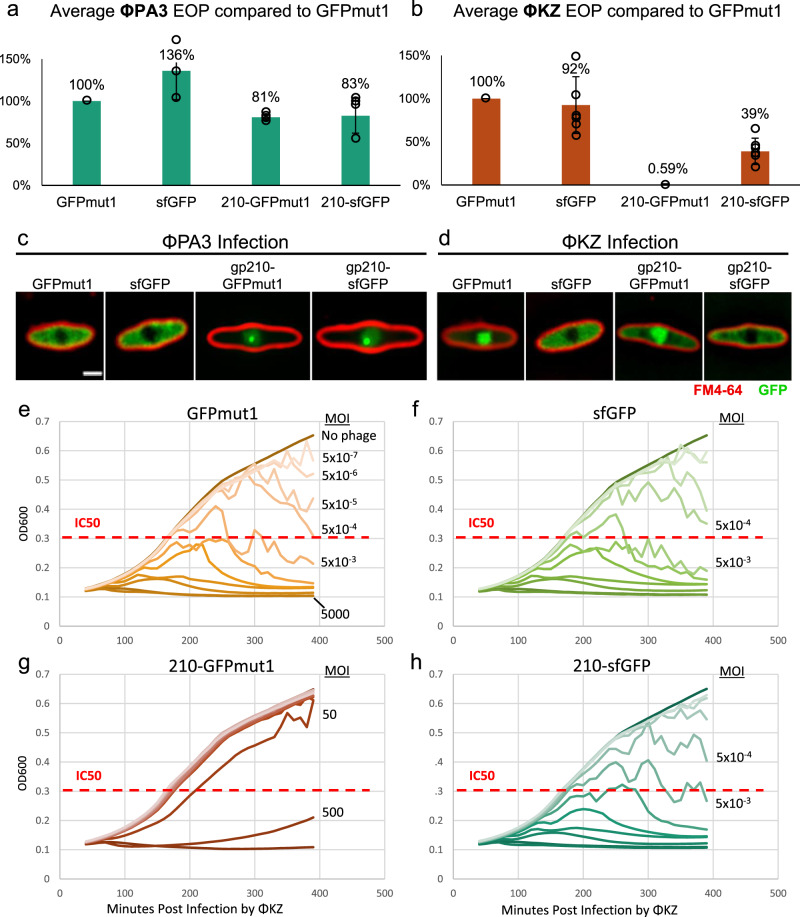
A nuclear incompatibility determinant impairs phage fitness. **a** Efficiency of plating (EOP) relative to GFPmut1 for ΦPA3 in cells expressing the indicated fusions with 1% arabinose. The number of independent replicates, *n*, equals 4 (GFPmut1), 3 (sfGFP), 4 (gp210-GFPmut1), and 4 (gp210-sfGFP). Error bars equal standard deviation. **b** EOP relative to GFPmut1 for ΦKZ in cells expressing the indicated fusions with 1% arabinose demonstrate a 99.4% decrease in viable ΦKZ with gp210-GFPmut1 in the nucleus. The number of independent replicates, *n*, equals 7 (GFPmut1), 6 (sfGFP), 6 (gp210-GFPmut1), 6 (gp210-sfGFP). Error bars equal standard deviation. **c**, **d** GFP fusions (green) and FM4-64 stained cell membranes (red). Individual data points are shown as circles. **c** GFPmut1 and sfGFP localize to the cytoplasm during ΦPA3 infections. gp210-GFPmut1 and gp210-sfGFP localize to the nucleus and form puncta during ΦPA3 infections. **d** sfGFP and gp210-sfGFP localize to the cytoplasm of ΦKZ infected cells. GFPmut1 and gp210-GFPmut1 localize to the nucleus of ΦKZ infected cells. **e**–**h** Determination of ΦKZ IC50 (red dotted line) for cells expressing control proteins GFPmut1 or sfGFP (**e**, **f**) or fusions to gp210 (**g**, **h**) as indicated by measuring cell growth (OD_600_) over 6.5 h of infection. Ten-fold serial dilutions of phage were added to cells resulting in a multiplicity of infection (MOI) ranging from 5000 to 5 × 10^−7^ as shown in (**d**). All growth curves represent an average of eight independent trials. For the sake of clarity, only the MOIs that indicate the IC50 are shown for (**f**–**h**). Source data are provided as a Source Data file.

Since we found an incompatibility factor in the cytoplasm, we reasoned that nuclear incompatibility factors might be important when hybrid nuclei between two distinct phages are formed, which occurs in ~25% of ΦKZ/ΦPA3 co-infections (Fig. 1d). We hypothesized that nucleases normally present in the nucleus may diverge between species and, if introduced into the opposing phage’s nucleus, would reduce its fitness. We identified five potential nucleases from ΦPA3 and imported them into the ΦKZ nucleus by taking advantage of our fortuitous finding that GFPmut1 and any proteins fused to it, are imported into the nucleus of ΦKZ but not ΦPA3 (Fig. 4c)^20^. In contrast, sfGFP does not alter protein localization and naturally resides in the cytoplasm (Fig. 4c), serving as a negative control^20^.

Of the five ΦPA3 proteins tested, only the putative endonuclease gp210 strongly inhibited ΦKZ reproduction when imported into its nucleus. Bioinformatically, gp210 has an HNH nuclease domain, but its specific biochemical activities are presently uncharacterized. When tagged with either GFPmut1 or sfGFP, and expressed from a plasmid with 1% arabinose, gp210 localized inside the nucleus of ΦPA3 without affecting the efficiency of plating (EOP) (Fig. 4a, c). In contrast, importing gp210-GFPmut1 into the ΦKZ nucleus decreased EOP by 99.4% compared to GFPmut1 alone (Fig. 4b, d). The cytoplasmically localized gp210-sfGFP control had a relatively small effect (~60% reduction) on ΦKZ EOP (Fig. 4b). When gp210-GFPmut1 was expressed at low levels with 0.1% arabinose, the EOP of ΦKZ was reduced by ~93% while cytoplasmic gp210-sfGFP was only reduced by 20% (Fig. S5A, B). This suggests a reduction in fitness resulting from importing an endonuclease from a closely related phage, representing another type of Virogenesis Incompatibility. Surprisingly, when we examined ΦKZ replication in cells expressing gp210-GFPmut1, we saw no obvious effect on the first round of phage replication as judged by nucleus size or lysis time (Fig. S5C–E).

We used bacterial growth curves to further quantify the effect of gp210 on phage fitness over multiple generations in liquid cultures. Ten-fold serial dilutions of phage were added to exponentially growing cells expressing either gp210-sfGFP or gp210-GFPmut1 at a low level from an uninduced arabinose promoter and then cell growth (OD_600_) was monitored over time. If the imported gp210-GFPmut1 impairs ΦKZ fitness, we expect an increase in the concentration of ΦKZ required to inhibit 50% of cell growth (IC50) after 6.5 h. We found the IC50 of ΦKZ for cells expressing control proteins sfGFP or GFPmut1 was a multiplicity of infection (MOI) of 5 × 10^−3^ (Fig. 4e, f). In comparison, the IC50 of ΦKZ for cells expressing gp210-GFPmut1 was an MOI of 500 (Fig. 4g). This shows that 100,000-fold more ΦKZ phage were required to inhibit the growth of cells by 50% when gp210-GFPmut1 was imported into the nucleus compared to the GFPmut1 control. As another control, the IC50 of ΦKZ for cells expressing gp210-sfGFP (which is localized in the cytoplasm) was an MOI of 5 × 10^−3^ (Fig. 4h) which is similar to the IC50 in the cells expressing control proteins sfGFP or GFPmut1.

These experiments demonstrate that gp210 is a native nuclear protein of ΦPA3 that is naturally excluded by the ΦKZ nucleus and is incompatible with ΦKZ replication when imported. Such nuclear incompatibility factors can provide continued selection for the nucleus, thereby reinforcing Subcellular Genetic Isolation and consequent divergence of these two species.

## Discussion

The Biological Species Concept defines species as interbreeding populations that are reproductively isolated from other populations^21^. Speciation factors in eukaryotes establish reproductive barriers that allow genetic divergence and the formation of new species. Here we describe three viral speciation factors that we categorize into two distinct reproductive isolation mechanisms. Subcellular Genetic Isolation can act by reducing genetic exchange between viruses co-infecting a single host cell, as in the case of the phage nucleus that physically separates phage genomes. Virogenesis Incompatibility can act by limiting the production of viable viral particles and reducing viral fitness, as occurs with incompatible hybrid spindles or the presence of a nuclear incompatibility factor such as gp210. These general principles of Subcellular Genetic Isolation and Virogenesis Incompatibility provide an understanding of the underlying mechanisms of viral speciation. We calculated that these mechanisms are sufficient for nearly complete genetic isolation between these two phages. The probability of a successful hybrid was estimated by multiplying the effects of Subcellular Genetic Isolation due to the phage nucleus (25%), relative genome frequency due to competition (94%), and Virogenesis Incompatibility due to hybrid spindles (50%) and a nuclear incompatibility factor (0.59%). While we cannot discern from these data if any of these factors caused the speciation of the two phages, we have demonstrated that they significantly reduce the chance of obtaining a successful hybrid to 0.07% under ideal circumstances where two diverging phages simultaneously infect the same cell. These results show Subcellular Genetic Isolation and Virogenesis Incompatibility lead to a limitation of gene flow, and together with previous work, directly support the application of the Biological Species Concept to viral speciation.

In line with other speciation mechanisms like Dobzhansky-Muller genetic incompatibilities, we predict that each of these mechanisms will have a greater impact on isolation with increased evolutionary divergence^22^. As the nucleus and spindles diverge, they are likely to accumulate functional and structural differences that result in increasingly defective configurations when they co-assemble. When mutations accumulate between diverging strains, evolving nucleases that are beneficial to one phage can become detrimental to a divergent phage. As shown in Fig. S2, when strains diverge in their competitive fitness they are less likely to recombine. Taken together, we expect that these mechanisms will favor genetic exchange between closely related strains, and create increasingly reinforced barriers between divergent strains.

These evolutionary principles likely apply to many viruses and help to explain their rapid evolution and diversity. Herpes virus has been shown to form spatially separated replication compartments (Subcellular Genetic Isolation) that limit genetic exchange but recombination occurs when these compartments coalesce^18^. In comparison, the phage nucleus also limits genetic exchange of viral genomes regardless of intracellular proximity. The phage nucleus, which may have evolved to provide protection against host defenses^14,15,23,24^, is likely widespread since it has been observed during infection of *Serratia* with phage PCH45^23^, and we have identified shell homologs in many distinct phage families infecting a diverse range of hosts including *Salmonella, Ralstonia, Cronobacter, Erwinia, Vibrio*, and *E. coli*. The PhuZ tubulin that makes up the spindle is also conserved and the genetic divergence that has occurred between the PhuZ proteins of ΦPA3 and ΦKZ results in Virogenesis Incompatibility that further limits exchange and drives speciation. Virogenesis Incompatibility could conceivably occur for any virus through the co-assembly of divergent proteins that make up any macromolecular structure (including virion structural proteins) required for phage propagation. These are the first described examples of intracellular speciation factors for any virus, demonstrating that even viruses evolve traits that facilitate reproductive isolation. This discovery suggests that all domains of life, including viruses, are challenged to optimize genetic exchange by way of speciation in order to enhance the supply of adaptive variation while minimizing the influx of incompatible genes.

## Methods

### General methods

*Pseudomonas aeruginosa* strain PAO1 was grown on LB agar containing 10 g Bacto-Tryptone, 5 g yeast extract, 5 g NaCl, and 15 g agar in 1 L ddH2O and incubated at 30 °C. Lysates of phages ΦPA3 and ΦKZ were prepared by infecting 10^9^ cells of *P. aeruginosa* strain PAO1 with phage (~10^8^ pfu/ml) at room temperature for 15 min and then adding 5 ml of 0.35% LB top agar onto an LB plate. Following overnight incubation at 30 °C, plates were flooded with 5 ml SM phage buffer and incubated at room temperature for 4 to 6 h. Phage lysates were harvested, centrifuged at 21,130 × *g* for 10 min and stored at 4 °C.

### Plasmid and strain constructions

*Pseudomonas aeruginosa* strain PAO1-K2733 was used for all experiments. Plasmids were introduced into strain PAO1-K2733 by electroporation and selecting on LB containing 15 µg/mL gentamycin. The vector pHERD-30T^14–16^ was used to express all GFP fusions. Plasmids expressing sfGFP-ΦPA3PhuZ, sfGFP-ΦKZPhuZ, sfGFP-ΦPA3PhuZ(D190A), sfGFP- ΦPA3shell and mCherry-ΦKZshell were previously described^14–16^. To construct a co-localization construct of ΦPA3 shell and ΦKZ shell, the pHERD-30T backbone with mCherry-ΦKZshell was first amplified using primers oVC701 and oVC406 by PCR from plasmid pMAC011^15^. The GFPmut1 gene tagged at the N-terminus of ΦPA3shell gene in plasmid pVC077 was replaced by sfGFP^15^. The insert of sfGFP-ΦPA3shell was then made by PCR amplification using primers oVC403 and oVC702 from the resulting plasmid. These 2 amplicons were assembled together by NEBuilder^®^ HiFi DNA Assembly Cloning Kit (New England Biolabs) resulting in a co-localization construct of sfGFP-ΦPA3shell and mCherry-ΦKZshell (pMAC082). We then performed the same cloning strategy to construct a co-localization plasmid of ΦPA3 PhuZ and ΦKZ PhuZ. The primers oVC701 and oVC406 were used to amplify the pHERD-30T backbone with mCherry-ΦKZPhuZ from plasmid pVC116 while the primers oVC403 and oVC702 were used to amplify the insert of sfGFP-ΦPA3PhuZ from plasmid pVC028. These 2 amplicons were then assembled using NEBuilder^®^ HiFi DNA Assembly Cloning Kit (New England Biolabs) resulting in a co-localization construct of sfGFP-ΦPA3PhuZ and mCherry-ΦKZPhuZ (pVC120). An untagged ΦKZPhuZ plasmid was made using oVC704 and oVC705 to amplify the linear amplicon of ΦKZ PhuZ linked with pHERD-30T from pVC029^15^. The linear amplicon was then circularized by ligase resulting in plasmid pVC121. Plasmids containing GFPmut1 and sfGFP were previously described^14–16^. gp210 was amplified from ΦPA3 lysate using primers oEB003 and oEB004. Plasmid pKN053 and pKN069 were linearized using oEB005 and oEB006. gp210 was assembled with each linearized plasmid using NEBuilder^®^ HiFi DNA Assembly Cloning Kit (New England Biolabs). See Tables 1 and 2 for lists of primers, plasmids, and strains. All plasmids were confirmed by DNA sequencing.

**Table 1:** Table of oligonucleotides used in this study.

Primer	Sequence (5′ – 3′)
oVC701	5′- GA TAAGAAGGAGATATACATAC atggtgagcaagggcga -3’
oVC406	5′- GGTATGGAGAAACAGTAGAGA -3’
oVC403	5′- TCTCTACTGTTTCTCCATACC -3’
oVC702	5′- ATGTATATCTCCTTCTTATCAATTAATAGAAGCCCGAGCCT -3’
oVC704	5′- ATGATGTCTAAAGTAAAAACTCG -3’
oVC705	5′- GGGTATGTATATCTCCTTC -3’
oEB003	5′- AGAAGGAGATATACATACCC ATGGCTATAAACTTAAAGGA -3’
oEB004	5′- CCTGCAGCGGCCGCTCCGGA gatatcgctatctagtggga -3’
oEB005	5′- TCCGGAGCGGCCGCTGCAGG -3’
oEB006	5′- GGGTATGTATATCTCCTTCT -3’

**Table 2:** Table of plasmids and strains used in this study.

Insert	Backbone	Host	Plasmid	Strain	Experiment
sfGFP-ΦPA3shell-mCh-ΦKZshell	pHERD-30T	PA01-K2733	pMAC082	MAC084	Single/dual infection
sfGFP-ΦPA3PhuZ	pHERD-30T	PA01-K2733	pVC028	VC568	Single infection
sfGFP-ΦPA3PhuZ(D190A)	pHERD-30T	PA01-K2733	pVC032	VC570	Single infection
sfGFP-ΦKZPhuZ	pHERD-30T	PA01-K2733	pVC029	VC586	Cross infection
sfGFP-ΦKZPhuZ(D204A)	pHERD-30T	PA01-K2733	pVC034	VC590	Cross infection
Untagged ΦKZPhuZ	pHERD-30T	PA01-K2733	pVC121	VC614	Cross infection
mCh-ΦKZPhuZ-sfGFP-ΦPA3PhuZ	pHERD-30T	PA01-K2733	pVC120	VC609	Single infection
GFPmut1	pHERD-30T	PA01-K2733	pKN053	KN381	Single infection
sfGFP	pHERD-30T	PA01-K2733	pKN069	KN529	Single infection
ΦPA3gp210-GFPmut1	pHERD-30T	PA01-K2733	pMAC185	EB003	Cross infection
ΦPA3gp210-sfGFP	pHERD-30T	PA01-K2733	pEB001	EB007	Cross infection

### Live cell fluorescence microscopy

Single cell infections of *P. aeruginosa* were visualized using fluorescence microscopy^14–16^. Briefly, 1% agarose pads containing 25% LB, 2 µg/ml FM 4-64, and 1 µg/ml DAPI were inoculated with 5 μL of cells (~1 × 10^8^ cfu/ml) allowed to grow at 30 °C in a humidor for 3 h and then infected with 10 µl of a high titer (10^12^ pfu/ml) phage lysate. For dual infections, lysates of ΦPA3 and ΦKZ were mixed at an equal ratio prior to infections. At desired time point after phage infection, a coverslip was put on the slide and cells were imaged using a DeltaVision Spectris Deconvolution microscope (Applied Precision, Issaquah, WA, USA). Images were further processed by the deconvolution algorithm in DeltaVision SoftWoRx 6.5.2 Image Analysis Program, and analyzed using Fiji 1.53c software. For time-lapse microscopy, infections were established on agarose pads (25% LB, 1% agarose, 0.1% arabinose) without stains. At 30 min post-infection, locations for taking time-lapse images were identified, and at 45 min post-infection, images were captured every 10 min for four hours. The cells from the resulting images were counted and the number of infected cells was divided by the total number of starting cells to determine the percentage of cells that were lysing over the course of the four hours. The percentages were averaged and plotted for both conditions.

### Fluorescence recovery after photobleaching (FRAP)

Cells containing sfGFP-ΦPA3PhuZ filaments were prepared as indicated for fluorescence microscopy and photobleach using a laser (QLM module, API) for 0.05 s at 31% power. Images were collected every 2 s for 1 min by an Applied Precision/GE OMX V2.2 Microscope. Images were deconvolved with DeltaVision SoftWoRx 6.5.2 and analyzed by Fiji 1.53c.

### IC50 growth curves

We determined the concentration of phage required to inhibit 50% cell growth over 6 h as a measure of phage fitness. 5 mL cultures of *P. aeruginosa* in liquid LB containing 15 μg/mL gentamycin were grown to an OD_600_ of approximately 0.6 to 0.8 and then diluted to an OD_600_ of 0.1. 5 μL of ten-fold serial dilutions of a ΦKZ lysate with a titer of 10^12^ pfu/ml were added to each well of a 96-well plate containing 100 μL of diluted bacterial culture. The 96-well plates were shaken for an initial 40 min at 30 °C in a microplate reader after which OD_600_ measurements were taken every 10 min for 6 h, with continuous shaking at 30 °C between the timepoints. The resulting OD_600_ values were averaged and plotted as a growth curve. Fusions were expressed from a plasmid at very low levels without arabinose induction.

### Efficiency of plating titers

To compare the efficiency of phage plaque formation on different strains, 10^8^ cells were infected with 10 fold serial dilutions of phage lysate at room temperature for 15 min and then 5 ml of 0.35% LB top agar was added and poured onto an LB plate. Plaques were counted after 24 h of incubation at 37 °C and the efficiency of plating calculated as the ratio of the test sample to the control. Protein expression was achieved with the indicated fusions by inducing with either 1.0% or 0.1% arabinose^14–16^.

### Co-infection shell analysis

Fiji image analysis program was used for image analysis. A total of 89 co-infected cells containing both shells were included in the analysis. For size and DAPI intensity analysis of the shell, a polygon was drawn over the shell to measure the area and mean DAPI intensity of the shell. Then, the mean DAPI intensity was subtracted by the background DAPI intensity of the corresponding image yielding the absolute DAPI intensity of the shell for the analysis. For shell position analysis, the distance between the center of the shell and the mid-position of the cell length was measured. Then, the distance was normalized by the corresponding cell length creating the fraction of the cell length for data visualization.

### Statistics and reproducibility

All experiments, including microscopy, were repeated at least three times. Representative microscopy images are shown. Standard deviations were calculated for phage titer experiments.

### Reporting summary

Further information on research design is available in the Nature Research Reporting Summary linked to this article.

## Supplementary information

Supplementary Information

Reporting Summary

## Data Availability

The authors declare that all the data supporting the findings of this study are available within the paper and its Supplementary Information files or from the corresponding author upon reasonable request. Source data are provided within this paper. Raw images uploaded to: https://data.mendeley.com/datasets/sp93zcmvfs/1. Source data are provided with this paper.

## Source data

Source Data
