# Supplementary material for: Viral speciation through subcellular genetic isolation and virogenesis incompatibility
Source: Nat Commun. 2021 Jan 12;12:342. doi: 10.1038/s41467-020-20575-5 (PMC7804931; doi:10.1038/s41467-020-20575-5)
Supplement: Supplementary file 1 — Supplementary Information [file 41467_2020_20575_MOESM1_ESM.pdf]

Supplementary Information

a

mpi	ΦPA3	ΦKZ
20	24%	19%
50	21%	18%

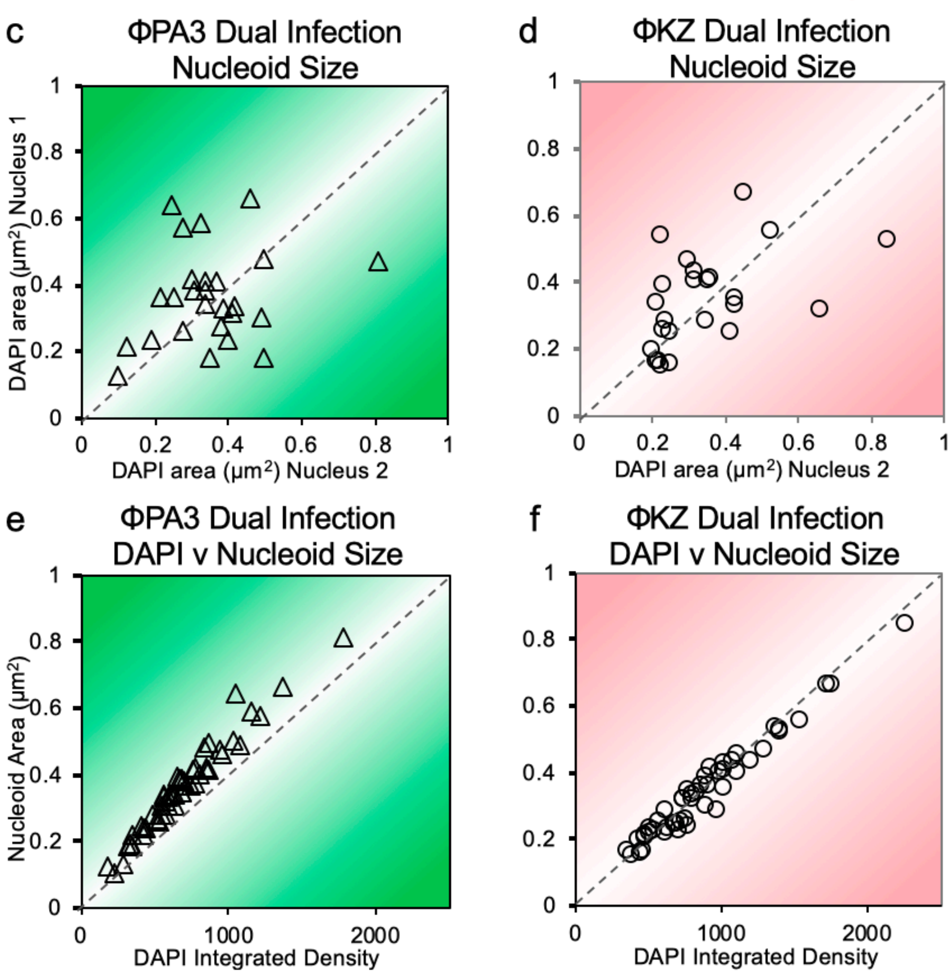

Figure S1. Viral nucleoid size and shell size of single species dual infections. A) Table of single-species dual infection percent per total infected cells, observed at 20 or 50 minutes post infection (mpi). For ΦKZ, n=368 (20mpi) and n=307 (50mpi). For ΦPA3, n=156 (20mpi) and n=142 (50mpi). B) Uninfected *P. aeruginosa* expressing GFP-PA3shell (green) and mCherry-KZshell (red) form diffuse fluorescence and foci. C, D) Nucleoid area as measured by area of DAPI staining and plotted for two nucleoids in single cell for ΦPA3 (C, n= 26 cells) and ΦKZ (D, n=25 cells) dual infections. The relative amount of nucleoid DNA was estimated by measuring integrated DAPI intensity and found to be proportional to nucleoid area for ΦPA3 (E, n=52) and ΦKZ (F, n=50). G) Average size of each shell during a co-infection (n=89).

Calculation of assortative mating frequency (Figure S2). One mechanism that can reduce the chance of cross-species phage recombination, even in the absence of protected shells, is through intracellular competition. Imagine a scenario where phage species infect a cell simultaneously and their genomes begin to replicate diffusely throughout the cytoplasm. If the two phages reproduce equally well, then they will achieve equal frequencies intracellularly and each phage species will have an equal probability of recombining with conspecifics or the other species. Alternatively, if one phage is able to allocate more resources and replicate more DNA, then the probability of cross-species recombination will be reduced since the majority of the recombinations will happen between conspecific genomes of the dominant species. This can be shown mathematically with calculations that are similar to the Hardy Weinberg equation from population genetics. If a cell were infected by two phages and these phages vary in their growth rates, then their frequencies would shift from 0.5 and 0.5 to p and q. Additionally, if it is assumed that most recombination between genomes occurs near the end of DNA replication when phage DNA is at its highest concentration, then p and q can be used to predict the relative frequencies of intraspecies versus interspecies recombination. The probability that recombination will occur between two P genomes is equal to the likelihood that two P genomes encounter each other, which is the product of their frequency in the cell, p^2. The probability of recombination between two Q genomes is q^2. The total amount of intraspecies recombination will be $p^2 + q^2$. The probability of P recombining with Q is pq, and the probability of Q recombining with P is qp. Therefore, $2pq$ is the probability of cross-species recombination. When $p = q$, then 50% of the recombination is interspecies and 50% is intraspecies. As the difference between the phages' ability to reproduce is magnified, their final frequencies will diverge, and interspecies recombination will become increasingly rare (Fig. S2).

We calculated 2pq for each individual co-infection based on DAPI staining intensity within each nucleus at 60 mpi and found the average 2pq value equaled 0.47. Therefore, the expected impact on the rate of recombination between PA3 and KZ based on genome frequency was a 6 percent reduction (Fig. S2).

This calculation makes many simplifying assumptions and additional factors could also contribute to genetic isolation. Among the additional factors we could conceive, all of them would reduce the potential for cross-strain recombination, making our estimations conservative. For example, our calculation assumes that the genetic differences between the strains does not impact the potential for them to recombine. However, it is well known that as DNA sequences accumulate more genetic mutations, they will be less likely to recombine (*1*). Therefore, the probability of recombination will be the product of the potential for two genomes to encounter each other within a cell, as we have computed here, and how similar the genomes are to one another. Another factor that would also reduce the potential for cross-strain recombination is the timing of infection. The greater the time lag between infections, the less likely the different strains will recombine. There are many other mechanisms that could reduce recombination. Our goal was to show that a simple ecological mechanism, such as resource competition, can impact inter-strain recombination and thus reproductive isolation. To the best of our knowledge, the effects of intracellular viral competition on recombination had not been previously considered. Given that intracellular competition should arise between any two strains infecting the same cell, we predict that this mechanism could have a significant impact on establishing genetic isolation in the virosphere.

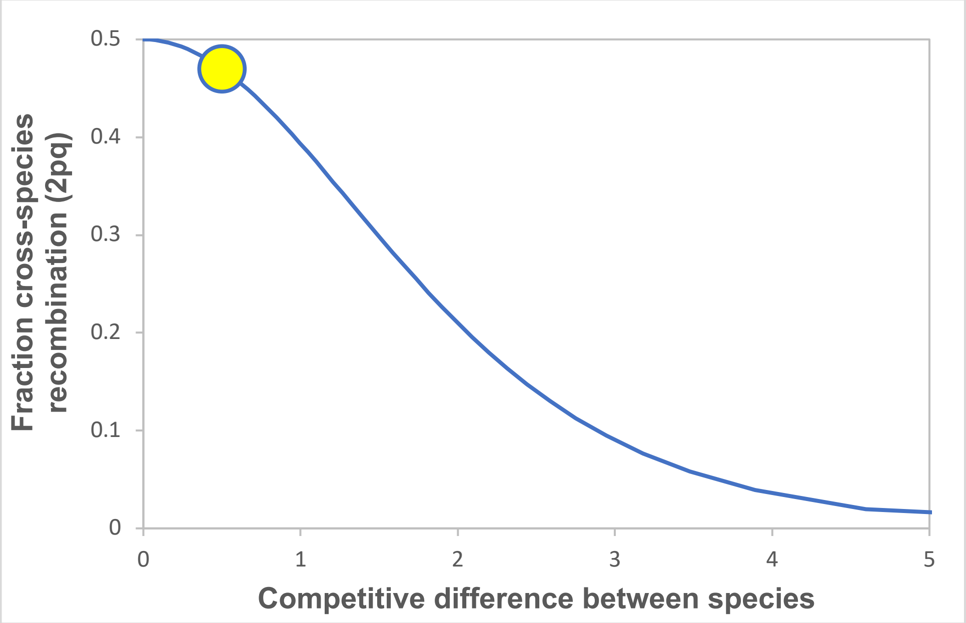

Figure S2. Fraction of cross-species recombination varies with competitive imbalance between species. Competitive differences were calculated using a modified selection rate equation, which computes the difference in Malthusian growth parameters over a period of time (*t*). Here, *t* will equal one round of infection and thus factors out of the equation. Competitive difference = ln(p/0.5) – ln(q/0.5). The yellow circle indicates the value calculated for ΦPA3 and ΦKZ based on their relative DNA concentrations.

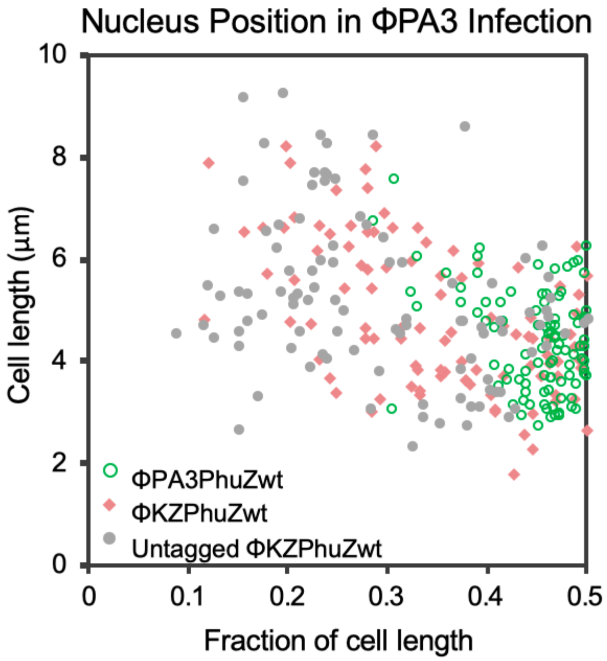

Figure S3. Nucleus positioning of ΦPA3-infected cells showing that the nuclei are mispositioned during cross-infections. ΦPA3 nucleus position is displayed as fraction of cell length for cells expressing wild type sfGFP-PA3PhuZ (green, n=100), or wild type sfGFP-KZPhuZ (red, n=100), or untagged KZPhuZ (grey, n=100). Source data are provided as a Source Data file.

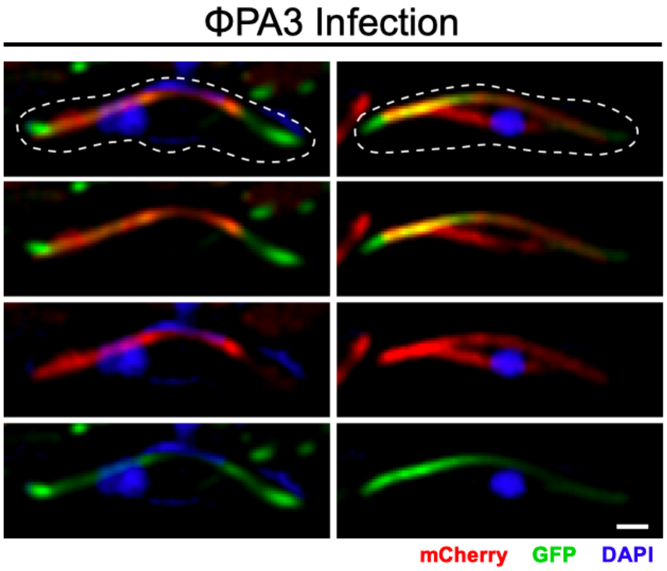

Figure S4. Two examples of nonfunctional hybrid spindles formed through cross species mixing of PhuZ monomers. Simultaneous expression of sfGFP-PA3PhuZ (green) and mCherry-KZPhuZ (red) results in hybrid filaments (yellow) and mispositioning of the ΦPA3 nucleus (blue).

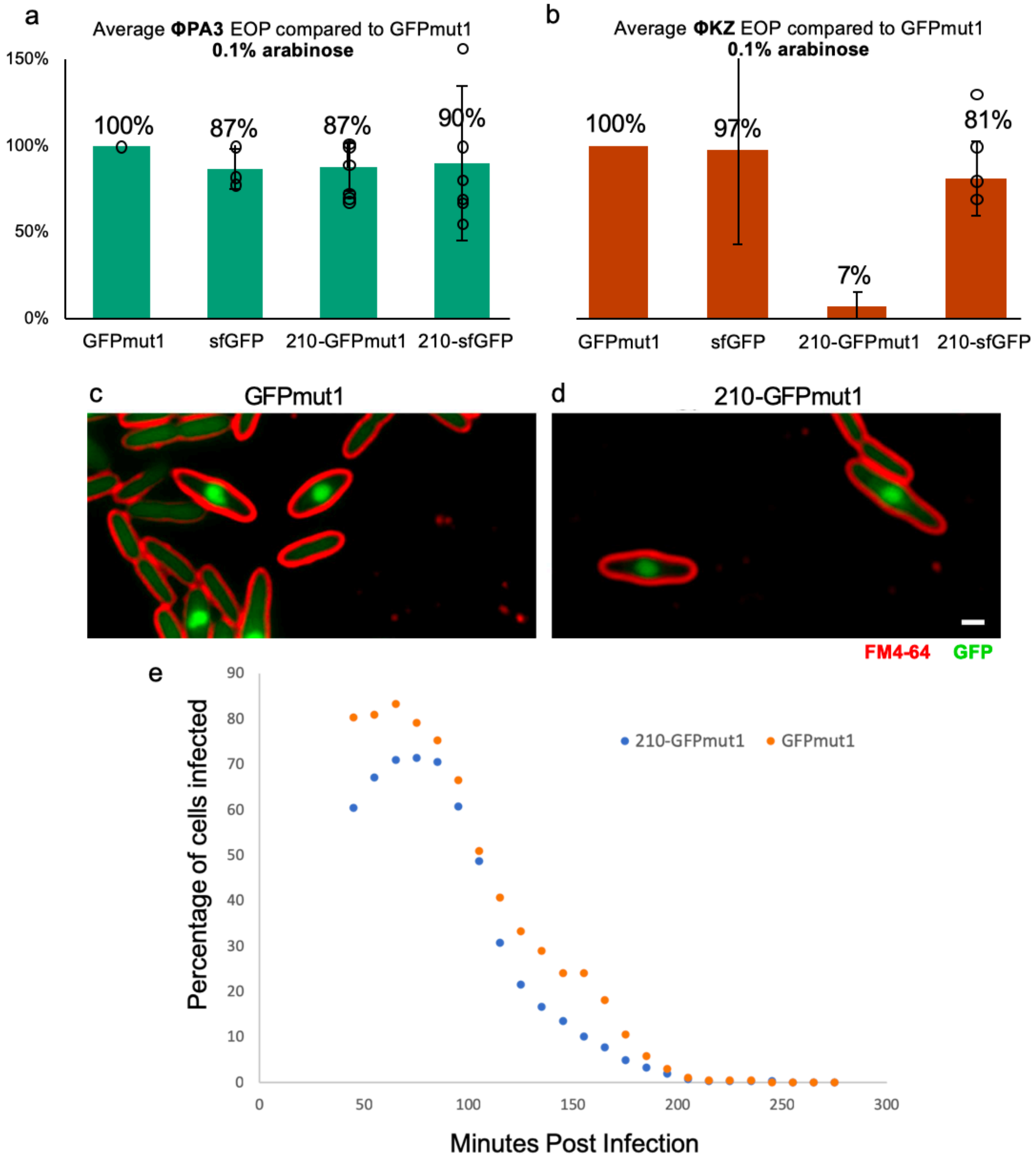

Figure S5. A) Efficiency of plating (EOP) relative to GFPmut1 for ΦPA3 in cells expressing the indicated fusions with 0.1% arabinose. The number of independent replicates, n, equals 7 (GFPmut1), 3 (sfGFP), 7 (gp210-GFPmut1), and 6 (gp210-sfGFP). Error bars equal standard deviation. B) EOP relative to GFPmut1 for ΦKZ in cells expressing the indicated fusions with 0.1% arabinose demonstrate a 93% decrease in viable ΦKZ with gp210-GFPmut1 in the nucleus. The number of independent replicates, n, equals 19 (GFPmut1), 6 (sfGFP), 19 (gp210-GFPmut1), 18 (gp210-sfGFP). Individual data points are shown as circles for n<10. Error bars equal standard deviation. C, D) Fields of cells at 70 mpi showing that *P. aeruginosa* cells expressing gp210-GFPmut1 (C, n= 3 replicates with 177 total cells) or a GFPmut1 alone (D, n= 5 replicates with 172 total cells) both form large, centrally positioned nuclei. GFP (green), cell membranes stained with

FM4-64 (red). ΦKZ replication in cells expressing gp210-GFPmut1 appears normal during a single round of infection. E) The percentage of phage cells infected, as indicated by the presence of a nucleus, decreases over time as cells lyse at the end of the infection cycle. The timing of cell lysis after phage infection was measured in time-lapse microscopy. gp210-GFPmut1 (blue circles) or a GFPmut1 alone (orange circles). Cells were infected and followed for 4.5 hours.

References

1. V. M. Watt, C. J. Ingles, M. S. Urdea, W. J. Rutter, Homology requirements for recombination in Escherichia coli. *Proc Natl Acad Sci U S A* **82**, 4768-4772 (1985).
